# The Impact of Traditional Cardiovascular Risk Factors on Cardiovascular Outcomes in Patients with Rheumatoid Arthritis: A Systematic Review and Meta-Analysis

**DOI:** 10.1371/journal.pone.0117952

**Published:** 2015-02-17

**Authors:** Leena R. Baghdadi, Richard J. Woodman, E. Michael Shanahan, Arduino A. Mangoni

**Affiliations:** 1 Department of Family and Community Medicine, King Saud University, Riyadh, Saudi Arabia, Department of Clinical Pharmacology, School of Medicine, Flinders University, Adelaide, Australia; 2 Flinders Centre for Epidemiology and Biostatistics, School of Medicine, Flinders University, Adelaide, Australia; 3 Department of Rheumatology, School of Medicine, Flinders University, Adelaide, Australia; 4 Department of Clinical Pharmacology, School of Medicine, Flinders University, Adelaide, Australia; University of Perugia, ITALY

## Abstract

**Background:**

Rheumatoid arthritis (RA) is known to increase the risk of cardiovascular (CV) disease. However, the individual impact of traditional CV risk factors in RA is unknown.

**Objective:**

To assess the strength of the association between individual CV risk factors and rate of either myocardial infarction (MI), combined CV morbidity (MI, angina pectoris, heart failure, stroke, and peripheral arterial disease (PAD)) or CV mortality in RA patients.

**Methods:**

RA studies reporting traditional CV risk factors [hypertension, type 2 diabetes (T2D), smoking, hypercholesterolaemia, obesity, and physical inactivity] as exposures and MI, CV morbidity (MI, angina, heart failure, stroke, and PAD combined) or CV mortality alone as outcomes were searched until March 2013 using MEDLINE, Scopus and Cochrane. Meta-analyses combined relative risk (RR) estimates from each study where either the RR and 95% confidence intervals or where raw counts were available.

**Results:**

Ten studies reporting sufficient data for inclusion into meta-analyses were identified. Relevant data was available for each risk factor and MI and CV morbidity but no studies reported on CV mortality. Risk of MI increased in RA patients with hypertension (RR 1.84, 95% CI 1.38, 2.46) and T2D (RR 1.89, 95% CI 1.36, 2.63). CV morbidity increased with hypertension (RR 2.24, 95% CI 1.42, 3.06), T2D (RR 1.94, 95% CI 1.58, 2.30), smoking (RR 1.50, 95% CI 1.15, 1.84), hypercholesterolaemia (RR 1.73, 95% CI 1.03, 2.44) and obesity (RR 1.16, 95% CI 1.03, 1.29) but not with physical inactivity (RR 1.00, 95% CI 0.71, 1.29).

**Conclusion:**

Hypertension, T2D, smoking, hypercholesterolaemia and obesity increased CV risk in patients with RA. These results highlight the importance of managing CV risk factors in RA, similarly to non-RA patients.

## Introduction

Rheumatoid arthritis (RA) is a chronic inflammatory autoimmune disease complicated by progressive joint destruction [1]. RA is often characterized by extra-articular manifestations mainly affecting cardiovascular, immune, respiratory, and renal systems. One of the most common and, indeed, serious complications is cardiovascular disease (CVD). A 48% and 60% excess risk of CV morbidity [2,3] and mortality [4] respectively, have been reported in RA patients. The association between RA and CVD has been linked to a pro-inflammatory state [5–8]. However, this does not seem to be the only mechanism involved as an increased CV risk is still present despite the availability of well-established anti-inflammatory medications in this patient group [9].

Traditional risk factors such as hypertension, type 2 diabetes (T2D), smoking, hypercholesterolaemia, obesity and physical inactivity is likely to explain at least some of the excess CV risk in RA patients, similarly to what has been extensively reported in the general population [10–14]. However, whether the relative impact of individual CV risk factors on CV risk, beyond that caused by RA alone, is similar to the general population is still unclear. Studies on hypertension, T2D, smoking, hypercholesterolaemia, obesity and physical inactivity have provided conflicting results. Few studies have found an increased CV risk in hypertensive RA patients in contrast to the general population [12,15–18]. There is no conclusive evidence on the presence, and magnitude, of an association between T2D and CV morbidity in RA. While some authors argue that the increased CV risk observed in RA patients is unrelated to the presence of T2D [11,18,19], others have documented that its presence increases CV risk [12,14,16,20,21]. Similarly, although smoking is well established as a CV risk factor, its potential impact in RA patients remains unclear [2,12,14,20,22–24] with reports of weak [25] or non-significant associations between smoking and CV risk in RA [12,18]. The impact of hypercholesterolaemia [12,14,16,26–32], obesity [15,33–35] and physical inactivity [13,36] on CV morbidity in RA have been contrasting with some decreases in risk with the presence of a risk factor; a phenomenon known as ‘risk factor paradox’. These paradoxes may arise due to several different forms of selection bias which can occur particularly in rheumatic disease research and have been termed ‘index event bias’ or ‘collider stratification bias’[37–40] and occur as a result of conditioning on a common effect (the index event). As a result, instead of reducing bias due to confounding, a spurious (perhaps negative) association may be induced. This usually occurs when multiple independent risk factors lead to both the development of the disease itself (the “index event” i.e. RA) and the disease sequelae (CV morbidity or mortality). In addition to index event bias, differential loss to follow-up, differential depletion of susceptible participantsand immortal time bias (differential misclassification of pre-exposure periods) are all forms of selection bias threatening the internal validity of the findings reported in rheumatic diseases research.

These issues notwithstanding, the aim of this systematic review and meta-analysis was to investigate the relative impact of individual traditional CV risk factors on CV morbidity and mortality among patients with RA.

## Material and Methods

### Searching methodology

A literature search was conducted for articles on the impact of traditional CV risk factors on CV morbidity and mortality among patients with RA. Pre-Medline, Medline, Scopus and Cochrane databases were searched until March 2013; the Premedline and Medline databases were searched using PubMed. Articles were identified by using controlled vocabulary terms (MeSH terms) as well as keywords (S3 Materials). Hand searching the citation lists of relevant articles was performed to look for additional papers.

### Study selection and patient outcomes

The exposure of interest was the presence or absence of risk factors in RA patients. The outcomes of interest were MI, combined CV morbidity (incidence of combined CV morbidity including MI, angina pectoris, heart failure, stroke, and peripheral arterial disease), and CV mortality.

Studies were included for inclusion in the meta-analyses if: (i) the diagnosis of RA in adult patients (≥ 18 years) was made according to current guidelines or by a rheumatologist; (ii) traditional CV risk factors (hypertension, T2D, smoking, hypercholesterolaemia, obesity, and physical inactivity) were assessed; (iii) the assessed outcomes included either myocardial infarction (MI), combined CV morbidities and/or CV mortality; (iv) either the raw count data or the estimated relative risk (RR) and 95% confidence interval of risk factors on CV morbidity were reported. Relevant studies were excluded if (i) the above inclusion criteria were not fulfilled; (ii) no information about clinical CV morbidity was available; (iii) no information about the effect of CV risk factors on clinical CV morbidity was available; (iv) the required data was not available.

### Data extraction

Data from each study was summarised in terms of: study design, participant characteristics, the assessed CV risk factors and outcomes, study quality score (Qi), and a summary of estimated effects.

### Quality scores of included studies

The different methodological approaches used across the studies required that the differences in study quality were accounted for. A reproducible and effective checklist is a feasible way to assess the quality of studies included in the meta-analysis, distinguishing between those with higher precision and reduced bias. Therefore, a generic checklist based on published studies [41,42] was used to assess the quality of selected studies and to calculate the study quality score (Qi). This checklist consisted of 14 questions evaluating internal validity, external validity and statistical analysis. Points awarded for each question were added to calculate the Qi score; high or low quality score was defined as Qi score of ≥10 or ≤ 9, respectively. Some of the questions were tailored to meet the study requirements (S1 Table). The balancing of key prognostic indicators affecting CV morbidity across exposure groups was considered in the checklist when creating a prognostic score (question 9 of checklist in S1 Table). The prognostic items included age, sex, hypertension, body mass index (BMI), diabetes, hypercholesterolaemia, smoking, family history of CVD, physical inactivity, duration of RA, and medications (folic acid, corticosteroids, and anti-rheumatic medications). A prognostic score of 1 was given to studies that balanced five or more of these items across comparison groups; a score of 0.5 was given to studies that balanced three or four items; a score of 0 was given if none, one or two of these items were balanced, or not documented in the study.

### Statistical analysis

Meta-analyses were performed to assess the association between exposure to CV risk factors and MI, and between exposure to CV risk factors and combined CV morbidity (MI, angina pectoris, heart failure, stroke, and peripheral arterial disease). For MI, where raw counts for exposed/non-exposed and event/non-event groups were available we used MetaXL software version 1.2 (www.epigear.com, Brisbane, Queensland, Australia). For combined CV morbidity, we first used raw counts where available to calculate effect sizes (RR) and confidence intervals (CI) using MetaXL and then combined these with studies that reported effect sizes (RR) and CI only using the user-written “metan” command for STATA software (version 13.0, StataCorp LD, College Station, Texas, USA).

Statistical methods for testing heterogeneity such as Q-statistic and its variants have low statistical power. Therefore vigilance, common sense and prior biological knowledge are required when synthesizing the results of different studies [43]. Statistical heterogeneity was anticipated across different study groups if tau-squared was >0 and/or the Q-statistic was significant at a p < 0.1 [43].

A sub-group analysis was performed for the effect of hypertension on combined CV morbidities to examine the possible modifying effects of various patient characteristics including age, disease duration, year of publication, and type of treatments. Subgroups were defined by the mean age of RA patients (≤55 years or >55 years), mean disease duration (<5 years or ≥ 5years), year of publication (before or in 2007 or after 2007), and type of current treatment (using methotrexate alone or methotrexate with other disease modifying anti-rheumatic drugs).

No formal funnel plot analysis was conducted as there were less than ten studies included in each meta-analysis [44].

## Results

### Search and screening

**Fig 1:**
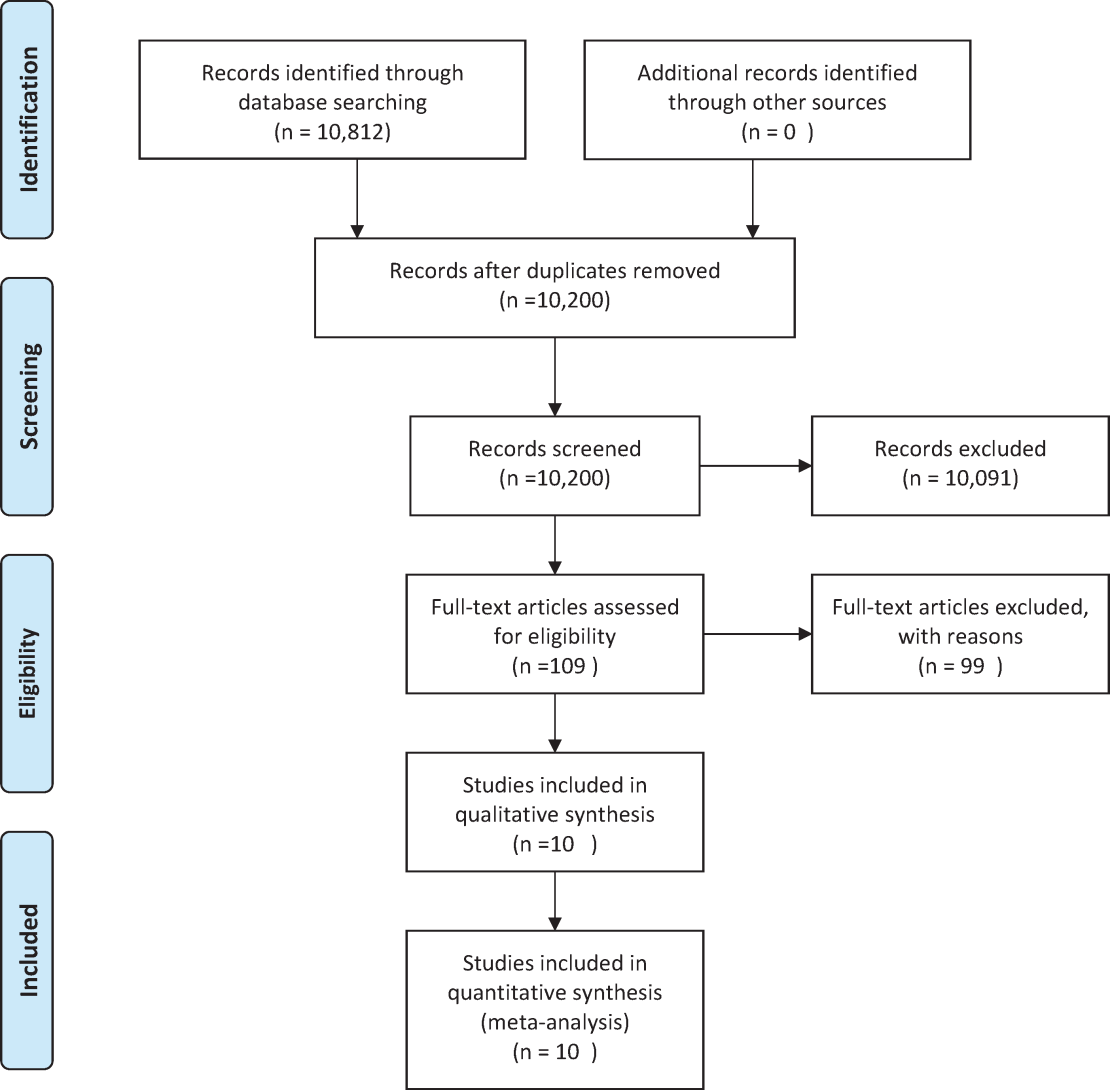
Flow diagram of study selection.

There were 10,812 studies identified through database search. After removing duplicates, there were 10,200 records published between 1947 and March 2013. The abstracts of these studies were screened and 10,091 reports were excluded, leaving 109 records (Fig. 1). After evaluating the full-text documents of these 109 records, 99 studies were excluded for the following reasons (S2 Table): 21 did not fulfil the inclusion criteria, 44 had no information on clinical CV morbidity, 25 had no information about CV risk factors, three publications were multiple reports on the same sample population [24,45,46] and six studies had no required data [11,13,14,16,20,35]. Authors of these six studies were contacted several times with a request for relevant information. Authors of one study refused to participate, two authors were unable to provide the requested data and three did not respond, despite several attempts.

Therefore, a total of 10 published studies meeting the inclusion criteria contributed to the various meta-analyses [10,12,15,17,19,21,25,36,47,48]. Of these, raw counts for exposed/non-exposed groups and event/non-event groups were extractable from only five studies [10,15,21,36,47] and provided data on a total of 4,388 RA patients exposed to the following CV risk factors: hypertension (n = 1,879), T2D (n = 453) and smoking (n = 2,056). The remaining five studies, however, reported estimated relative risks (RR) and 95% CI for combined CV morbidity and all risk factors [12,17,19,25,48].

### Characteristics of studies and subjects

Table 1 describes specific information from the final 10 studies meeting the inclusion criteria and included in the meta-analyses. There was considerable variation in study design, age, RA duration, methodological quality and ascertainment of exposure and outcomes across the 10 studies. One study had a nested case-control design, seven were retrospective cohorts, one prospective cohorts and one was cross-sectional studies. Mean age of RA patients ranged from 50 [15] to 67 years [47]. Mean RA duration ranged from ≤ 1 year [12,48] to 17 years [21]. Importantly, methodological quality varied among studies with scores ranging from 7 [10] to 11[12,19,47]. Each study reported on at least one CV risk factor (hypertension, T2D, smoking, hypercholesterolaemia, obesity and physical inactivity) but no standard criteria were used when ascertaining exposure to CV risks factors [10] or in the selection of RA patients [21]. Nevertheless, all studies suggested an increased CV morbidity (MI, angina, heart failure, stroke, and PAD combined) with different risk factors (Table 1). Similarly, there was a trend towards increased CV mortality among RA patients in the majority of studies [10,12,15,17,19,25,48].

**Table 1:** Studies meeting the inclusion criteria and included in the quantitative meta-analysis (n = 10).

Reference	N	Design	Participants	Risk factors assessed	CV outcomes	Risk factors associated with CV risks (estimated effect size)	Quality score (Qi) out of 14
[47]	RA with MI = 41; RA without MI = 181	Nested case-control study	RA diagnosed according to the 1987 revised ACR criteria; random selection of controls from the PCS was done; Mean age: 67.5±10 years; Mean RA duration: 4.3±8.5 years	*Hypertension, *T2D, *Smoking status, Dyslipidaemia, Obesity	CV morbidity: either MI or unstable angina	**OR of MI**	11
						ǂ Hypertension: OR 2.41 (95% CI 1.14, 5.11)	
						ǂ T2D: OR 1.29 (95% CI 0.40, 4.14)	
						ǂ Smoking: OR 1.46 (95% CI 0.72, 2.97)	
						HDL: OR 0.50 (95% CI 0.09, 2.71)	
						Obesity: OR 1.09 (95% CI 0.99, 1.20)	
[21]	Total RA = 369	RCS	No diagnostic criteria for RA; Mean age: 57±14 years; Mean RA duration: 17±11 years	*Hypertension, *T2D	CV morbidity: coronary heart disease, MI and CHF	**OR of MI**	8.5
						ǂ Hypertension: OR 2.48 (95% CI 1.31, 4.70)	
						ǂ T2D: OR 3.75 (95% CI 1.50, 9.35)	
[10]	Total RA = 239 (Female = 196; Male = 43)	RCS	RA diagnosed according to the 1987 revised ACR criteria; Mean age: 56.3±15.7 years; Mean RA duration: 11.6±8.8 year	*Hypertension, *T2D, *Smoking, Hypercholesterolemia, Body weight	CV morbidity: MI and stroke; MI as separate CV outcome and as combined CV event; MI; Stroke. CV mortality: CV death due to MI, stroke or CHF	**RR of combined CV morbidity**	7
						Hypertension: RR 4.3 (95% CI 1.4, 13.2)	
						ǂ T2D: RR 2.62 (95% CI 0.83, 8.28)	
						ǂ Smoking: RR 0.75 (95% CI 0.18, 3.13	
						Hypercholesterolemia: RR 6.0 (95% CI 1.80, 20.70)	
						# Body weight: -	
[36]	Total RA = 4,363 (Female = 3,403; Male = 960)	Cross-sectional	RA diagnosed according to the 1987 revised ACR criteria; Mean age: 57±1 years; Mean RA duration: 11±9 years	*Hypertension, *T2D, *Smoking status, Hypercholesterolaemia, Obesity, Physical activity	CV morbidity: MI, angina, coronary disease and stroke	**HR of combined CV morbidity**	10.5
						Hypertension: HR 2.97 (95% CI 2.31, 3.83)	
						T2D: HR 2.09 (95% CI 1.50, 2.92)	
						Smoking: HR 1.60 (95% CI 1.25, 2.04)	
						Hypercholesterolaemia: HR 3.19 (95% CI 2.47, 4.13)	
						Obesity: HR 1.34 (95% CI 0.96, 1.86)	
						Physical inactivity: HR 1.00 (95% CI 0.75, 1.33)	
[15]	Total RA = 325 (Female = 250; Male = 75)	RCS	RA diagnosed according to the 1987 revised ACR criteria; Mean male age: 56±15 years; Mean female age: 50±15 years; Mean RA duration: 2 years	*Hypertension, T2D, Smoking, Hypercholesterolaemia, Obesity, Physical activity	CV morbidity: MI, angina pectoris, coronary disease, and stroke; CV mortality: coronary heart disease death	**HR of combined CV morbidity**	10
						Hypertension: HR 3.76 (95% CI 0.99, 15.06)	
						T2D: HR 1.09 (95% CI 0.20, 5.92)	
						Smoking: HR 2.02 (95% CI 0.35, 7.69)	
						Hypercholesterolaemia: HR 1.03 (95% CI 0.22, 4.75)	
						Obesity: HR 0.71 (95% CI 0.13, 3.85)	
						Physical inactivity: HR 2.53 (95% CI 0.31, 20.56)	
[19]	Total RA = 234; Non-RA = 5,158	RCS	RA diagnosed according to 1987 ACR criteria; O´RALE cohort was used; matched non-RA: SAHS cohort; Median age: 56 years (ranged 22–80)	Hypertension, T2D, Smoking, Hypercholesterolaemia, BMI	CV morbidity: MI or stroke or other arterial occlusive events or arterial revascularization procedures; CV mortality: CV deaths	**IRR of combined CV morbidity**	11
						Systolic blood pressure (per 15 mm Hg): IRR 1.18 (95% CI 1.03, 1.33)	
						T2D: IRR 2.28 (95% CI 1.65, 3.12)	
						Smoking: IRR 1.37 (95% CI 1.01, 1.83)	
						Hypercholesterolaemia: IRR 1.35 (95% CI 1.01, 1.82)	
						BMI (per 5 kg/m²): IRR 1.13 (95% CI 0.99, 1.28)	
[25]	Total RA = 603; Non-RA = 603	RCS	RA diagnosed by 1987 ACR criteria; matched non-RA cohort; randomly selected; Mean age: 58 years	Hypertension, T2D, Smoking, Hypercholesterolaemia, Obesity	CV morbidity: MI, CHF; CV mortality: CV death	**HR of combined CV morbidity**	9.5
						Hypertension: HR 1.97 (95% CI 1.24, 3.11)	
						T2D: HR 1.62 (95% CI 1.17, 2.24)	
						* Smoking: HR 1.32 (95% CI 0.97, 1.81)	
						Hypercholesterolaemia: HR 0.92 (95% CI 0.67, 1.26)	
						Obesity: HR = 1.27 (95% CI 0.93, 1.74)	
[12]	Total at entry = 700 (Male = 219; Female = 481);Total at end = 422 (Male = 141; Female = 301)	PCS	Early RA diagnosed by ARA criteria; patients records and self-reported questionnaire on co-morbidity and local rheumatologist follow up assessment were used; Mean age: 55.2±14.3 years; Mean disease duration: 3.3 months	Hypertension, T2D, Smoking, Dyslipidaemia, Obesity	CV morbidity: MI, stroke and peripheral vascular disease, Stroke/TIA, DVT/ PE; CV mortality: Fatal CV events	**HR of combined CV morbidity**	11
						Hypertension: HR 4.07 (95% CI 2.31, 7.16)	
						T2D: HR 2.89 (95% CI 1.30, 6.45)	
						# Smoking: -	
						Triglycerides level: HR 1.92 (95% CI 1.46, 2.52)	
						# Obesity: -	
[48]	Total RA = 211 (Male = 85; Female = 126)	RCS	Seropositive RA according to the 1958 revised diagnostic criteria for rheumatoid arthritis; Mean age for women: 50.6 years; Mean age for men: 53.7 years; RA duration ≤1 year; Patients selected from the only reference centre for rheumatology	Hypertension, T2D, Smoking	CV morbidity: MI, peripheral vascular disease and stroke, First CV event, Stroke/TIA, DVT/PE; CV mortality: Fatal CV events	**RR of combined CV morbidity**	10
						Hypertension: RR 2.48 (95% CI 1.48, 4.17)	
						# T2D: -	
						# Smoking: -	
[17]	Total RA = 606 (Male = 194; Female = 412)	RCS	Seropositive RA, classification number 71238 according to 8th edition, ICD-8, Swedish National Board of Health and Welfare, 1968; 1987 ARA criteria for RA; Mean age for women: 54 years; Mean age for men: 56 years; Mean RA duration: 12.5 years; patients selected from the only reference centre for rheumatology	Hypertension, T2D	CV morbidity: MI, peripheral vascular disease and stroke, DVT/PE, Cerebrovascular lesion/TIA; other CV events: peripheral arterial embolus and dissecting aorta aneurism; CV mortality: Fatal CV events	**RR of combined CV morbidity**	9.5
						Hypertension: RR 1.76 (95% CI 1.32, 3.35)	
						# T2D: -	

*Risk factors included in the meta-analysis because raw data was available

ǂ Calculated effect estimate (OR = ratio of odds of exposure among cases to odds of exposure among controls; RR = ratio of the probability of CV events in exposed group to the probability of the event in non-exposed group)

# Effect estimate could not be calculated as raw data was not available

ACR = American College of Rheumatology, RA = Rheumatoid Arthritis, ARA = American Rheumatism Association, RCS = Retrospective Cohort Study, PCS = Prospective Cohort Study, BMI = Body Mass Index, T2D = Type 2 Diabetes, CV = Cardiovascular, QUEST-RA = Questionnaires in Standard Monitoring of Patients with Rheumatoid Arthritis, MI = Myocardial infarction, CHF = Congestive Heart Failure, ICD-8 = International Classification of Diseases, eight revision, HR = Hazard ratio IRR = Incidence Rate Ratio, OR = Odd Ratio, RR = Relative Risk, CI = Confidence Interval; Qi = Study Quality Score

### Meta-analyses

No information was available on CV mortality, therefore only MI and combined CV morbidity (MI, angina pectoris, heart failure, stroke and peripheral arterial disease) were considered in the analyses (S3 Table)[10,12,15,17,19,21,25,36,47,48]. Two studies investigated MI as a separate CV outcome [21,47], one study described separate results for MI and for combined CV morbidity [10] and the remaining studies presented data for combined CV morbidity (S3 Table).

### Risk of myocardial infarction

**Fig 2:**
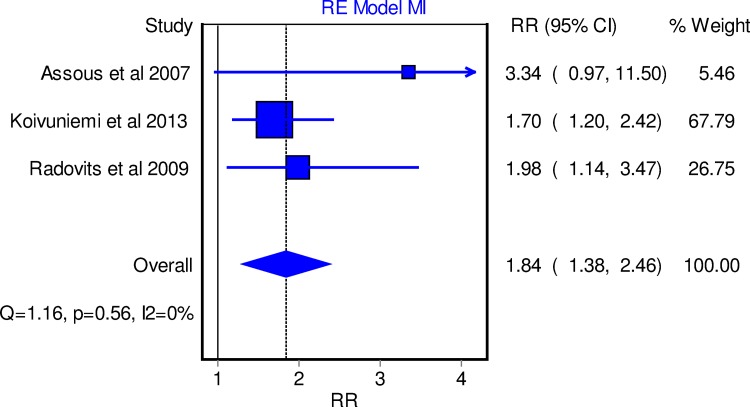
Forest plot depicting the relative risk of MI in hypertensive RA patients versus those without hypertension using random effect model.

**Hypertension.** Three out of 10 studies [10,21,47] showed an increased risk of MI in hypertensive RA patients. The RR was 1.84 (95% CI 1.38, 2.46) implying an 84% higher risk of MI among RA patients with hypertension compared with non-hypertensive RA patients (Fig. 2).

**Fig 3:**
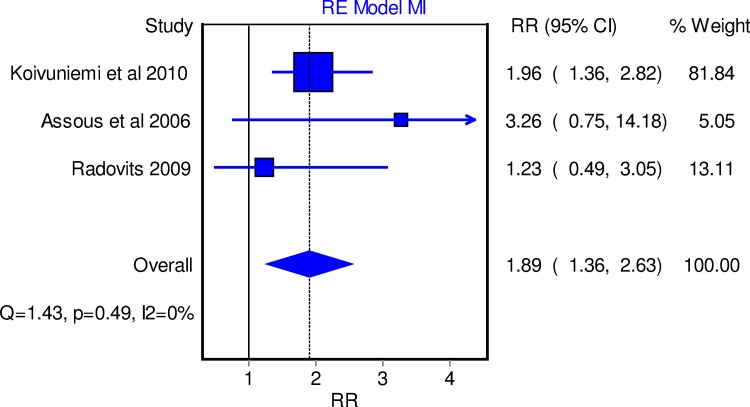
Forest plot depicting the relative risk of MI in diabetic RA patients versus those without T2D using random effect model.

**Diabetes.** Three out of 10 studies [10,21,47] showed an increased risk of MI in RA patients with T2D, leading to a combined RR of 1.89 (95% CI 1.36, 2.63) (Fig. 3).

### Risk of combined CV morbidity

**Hypertension.** Eight of the 10 studies [10,12,15,17,19,25,36,48] that assessed the risk of combined CV morbidity with hypertension showed an overall RR of 2.24 (95% CI 1.42, 3.06), implying that hypertensive RA patients are 2 times more likely to experience combined CV morbidity compared with non-hypertensive patients (S1 Fig.).

**Diabetes.** Five of the 10 studies [10,12,19,25,36] that assessed the risk of combined CV morbidity showed an excess risk in RA patients with T2D. Overall, RA patients with T2D were almost 2 times more likely to experience an event compared with non-diabetic patients (RR 1.94, 95% CI 1.58, 2.30) (S2 Fig.).

**Smoking.** Data from four out of 10 studies [10,19,25,36] examined the effect of smoking on the risk of CV morbidity. Overall, the RR was 1.50 (95% CI 1.15, 1.84) indicating a 50% increased risk of a CV event in smokers with RA compared to non-smoking RA patients (S3 Fig.).

**Hypercholesterolaemia.** Six out of 10 studies [10,12,15,19,25,36] that assessed the risk of combined CV morbidity showed an excess risk in RA patients with hypercholesterolaemia. Overall, RA patients with hypercholesterolaemia had a 73% increase in the incidence of combined CV morbidity compared with patients without this risk factor (RR 1.73, 95% CI 1.03, 2.44) (S4 Fig.)

**Obesity.** Data from four of 10 studies [15,19,25,36] that assessed the risk of combined CV morbidity with obesity showed an increased risk of combined CV morbidity, and an overall RR of 1.16 (95% CI 1.03, 1.29), implying that obese RA patients had a 16% increase in the incidence of combined CV morbidity compared with non-obese RA patients (S5 Fig.).

**Physical inactivity.** The two studies [15,36] that assessed the risk of combined CV morbidity showed no significant association between physical inactivity and combined CV morbidity in RA patients. Overall, the risk of combined CV morbidity in RA patients was similar in physically inactive and physically active RA patients (RR 1.00, 95% CI 0.71, 1.29) (S6 Fig.)

### Sub-group analysis

Table 2 shows the results of the sub-group analysis of hypertensive patients for combined CV morbidity. Hypertensive RA patients tended to have a higher risk of combined CV morbidity if: they were older (≥ 55years); duration of RA was shorter (< 5years); they were reported in studies published after 2007; and they were on combined anti-rheumatic medications especially if they were on biologics therapy. However, the trend of having higher risk of combined CV morbidity in hypertensive RA patients was retained.

**Table 2:** Sensitivity analyses of RA patients with hypertension and cardiovascular outcomes.

Parameters and Combined CV morbidity [RR RE model (95% CI)]
**Mean age of subjects (years)**
≤55 (n = 3): 2.04 (95% CI 1.24, 2.85)
>55 (n = 5): 2.35 (95% CI 1.20, 3.49)
**Mean duration of RA (years)**
<5 (n = 3): 2.88 (95% CI 1.72, 4.04)
≥5 (n = 3): 2.48 (95% CI 2.03, 3.21)
**Year of publication**
≤2007 (n = 4): 1.61 (95% CI 0.91, 2.32)
>2007(n = 4): 2.68 (95% CI 1.90, 3.47)
**Type of treatments**
MTX alone (n = 4): 2.18 (95% CI 0.50, 3.86)
Combined treatments (n = 4): 2.31 (95% CI 1.45, 3.16)
With biologics (n = 2): 2.99 (95% CI 2.23, 3.74)
Without biologics (n = 2): 1.43 (95% CI 0.71, 2.15)

n = number of studies involved in the analysis

CV: cardiovascular

## Discussion

Although there were inconsistencies in the literature reporting the impact of traditional CV risk factors in RA patients on MI and CV morbidity, this meta-analysis provides evidence for a significant negative impact of hypertension, T2D, smoking, hypercholesterolaemia and obesity in this population, with the magnitude of effects similar to that for the general population.

The role of traditional CV risk factors in the general population is well established [49–52]. Hypertension is a well-known modifiable risk factor [50] and the risk of CV morbidity can be reduced with a modest reduction in blood pressure. In the general population, an international case-control study estimated that people with hypertension were 91% more likely to develop MI (OR 1.91, 99% CI 1.74, 2.10) [50]. Hypertension is not only highly prevalent among patients with RA [53,54], but also an important predictor of CV events within this population [10,12,48,55]. This meta-analysis showed greater risk of both MI [10,21,47] and combined CV morbidity rates in RA patients with hypertension [10,12,15,17,19,25,36,48]. Compared with the general population, there was a similar increase in MI risk among RA patients with hypertension (RR 1.84, 95% CI 1.38, 2.46) (S4 Table). In line with our findings, it was recently reported that a systolic blood pressure increase of 20 mmHg in RA patients would result in 1,572 additional ischaemic heart disease events yearly (95% CI 1,024, 2,120) [56].

T2D is also a well-known risk for cardiovascular diseases. In the general population, T2D confers a 2–4 fold excess risk of CV morbidity [50,57–59]. Although the relationship between T2D and CV morbidities in RA has been questioned [2,18,19,25], results from five studies in this meta-analysis [10,12,19,25,36] showed that T2D increases CV risk in RA patients similarly to that in the general population. This is consistent with recent evidence from a prospective cohort study showing that T2D increased the incidence of combined CV morbidity compared with non-diabetic patients (HR 2.89, 95% CI 1.29, 6.45) [12]. The impact of T2D on MI risk tended to be meaningfully higher in the general population (OR 2.37, 99% CI 2.07, 2.71) than in RA patients (RR 1.89, 95% CI 1.36, 2.63) (S4 Table).

Although the prevalence of smoking in RA is well established [9], its impact on CV morbidity in RA have not yet been identified despite the established relationship in the general population [25,50]. In the INTERHEART study, current or former smokers in the general population were 2.3 times more likely to develop MI than non-smokers [50]. Interestingly, in RA patients, it has been claimed that there might be a weaker association between smoking and CV disease risk [25]; however, this result might be biased given the inherent risk of under reporting [60]. Most importantly, this smoking paradox might be caused by index event bias in which smoking is associated with the development of the disease itself (RA ‘index event’) and the disease sequelae (CV events). By conditioning on the index event of RA [40] (i.e. stratifying RA and non-RA patients) this may lead to a spurious association (reduced effect estimate) between the risk factor (smoking) and CV morbidities due to unmeasured or unknown confounders that are associated with both the index event and the events downstream of RA. Another explanation of the putative CV protective effects of smoking among RA patients is depletion of susceptible participant bias in which RA patients who smoke may die earlier but not from the outcome of interest. In a study showing a protective effect of smoking on CV events [25], RA patients who were more susceptible to CV complications related to smoking tended to die earlier of non-CV event outcomes. Although the effect of hypercholesterolemia on CV morbidity has not been well described, it was associated with higher combined CV morbidity among RA patients in this meta-analysis (RR 1.73, 95% CI 1.03, 2.44). On the other hand, the impact of body weight on CV morbidity showed a paradoxical relationship. Even though few authors argued that rheumatoid cachexia was associated with worse CV morbidity [33], others found no such association [34]. On the contrary, obesity was associated with increased CV events [19,25,35,36]. Our finding that obese RA patients had a 94% increase risk of combined CV morbidity supports this evidence. Compared to the general population, RA patients are usually less active [61]. As physical exercise improves both quality of life and physical function [62], encouraging RA patients to be more active has been suggested to be part of routine clinical care [63,64]. However, the two studies [15,36] in this meta-analysis found no significant association between physical inactivity and combined CV morbidity. This result should be interpreted with caution as one of the two studies in the meta-analysis had a cross-sectional design with a relatively short follow-up period [36]. Moreover, information about physical inactivity was based on self-report questionnaires.

Managing CV risk in RA patients is an emerging concept although little evidence exists regarding the efficacy and safety of specific treatment strategies [65]. It was traditionally assumed that the RA-associated pro-inflammatory state independently increased CV risk [5–8]. However, studies supporting this concept had a relative short follow-up period [5] and were often observational with a cross-sectional design. Assessing inflammatory markers at a single time point does not capture the cumulative burden of inflammation over time. Additionally, CV risk is still high despite the wide spread prescribing of anti-inflammatory medications in this population [9]. Therefore, it appears that other factors, e.g. traditional CV risk factors, might still have a role in this context.

Several factors potentially impacting on the pooled results were examined in sub-group analysis. A relatively higher risk of CV morbidity was observed in patients aged ≥55 years (RR 2.35 compared to those <55 years of age RR 2.04). This is consistent with the results of a recent prospective cohort study, showing that older RA patients had rapid disease progression and higher CV morbidity [66]. Interestingly, studies published after 2007 showed a higher impact of hypertension on CV morbidity (RR 2.68 compared to those studies published before or in 2007 RR 1.61). RA participants included in studies published after 2007 were followed between 2006 and 2008, which is known as Global Financial Crisis period. This economic crisis was linked to higher mortality rate in the general population [67]. It is possible that the observed high CV morbidity in studies published after 2007 was due to the impact of hypertension associated with financial stress; this imply that increased CV morbidity might be explained by the impact of CV risk factors. The widespread use of biologics in the last ten years is also a possible contributor to the variability in the observed effects of hypertension on CV morbidity. Although the use of biologics may more effectively reduce blood pressure among RA patients [68] our sub-group analysis of biologic versus non-biologic therapy on CV morbidity shows an increased risk amongst those using biologics. Clearly, further research is needed to corroborate these findings. Additionally, a trend towards increased CV risk was documented in patients with shorter disease duration (<5 years). Although this is in contrast with a report suggesting a higher CV risk with longer disease duration [65], recent evidence supports the concept of higher risk in the first few years of RA diagnosis [69]. It is possible that most RA patients are not appropriately treated with MTX during this critical period, i.e. within two years of diagnosis [66]. The importance of MTX was emphasized by results obtained from the sub group analysis; amongst studies assessing hypertensive RA patients treated with MTX alone, the risk of combined CV morbidity was slightly lower than all studies. Thus, MTX might have a protective effect in this population compared to other treatments for RA. Notably, hypertensive RA patients on treatment combination with biologics were approximately two times more likely as those patients on treatment combination without biologics to develop CV outcomes. Although some claim that biologics treatment, particularly tumour necrosis factor (TNF) blockers, reduces the risk of first CV event in RA patients [70], others have found no such association [71–75]. The reported protective effect of TNF blocker [70] may be biased as several important risk factors were not controlled for in this study. Furthermore, the protective effect of biologics might be explained by immortal time bias in which patients receiving biologics might have differentially had a shorter exposure period and longer pre-exposure assigned them than their control group counterparts. RA patients are initially started on non-biologics drugs, and therefore pre-exposure and follow-up time might be differentially classified between biologics and non-biologics group. Moreover, RA patients with complications such as infections may stop medication, causing differential loss to follow-up where selection bias occurs despite effective control for potential confounders [40]. Once again, further larger studies are required to investigate the impact of different RA treatment strategies on hypertension and cardiovascular risk in this group.

This systematic review and meta-analysis has some limitations. Studies included had different participant’s age at enrolment, RA duration and treatments type. These factors might be a potential confounders producing biased effect estimate. However, sub-group analysis was conducted and the trend of having higher risk of combined CV morbidity in RA patients was retained.

In conclusion, our meta-analysis indicates that despite the increased CV risk associated with RA in general, traditional CV risk factors such as hypertension, T2D, smoking, hypercholesterolaemia and obesity, independently increase the risk of CV morbidity in this patient population, and the magnitude of this increase appears similar to that observed in the general population. This suggests that a careful diagnosis and management of CV risk factors should be considered as important as the management of the symptoms of RA in mitigating the risk of CV morbidity and mortality amongst these patients.

## Supporting Information

S1 Fig Forest plot depicting the relative risk of combined CV events in hypertensive RA patients versus those without hypertension using random effect model. (TIFF)

S2 Fig Forest plot depicting the relative risk of combined CV events in diabetic RA patients versus those without T2D using random effect model. (TIFF)

S3 Fig Forest plot depicting the relative risk of combined CV events in RA patients who smoke versus non-smokers using random effect model. (TIFF)

S4 Fig Forest plot depicting the relative risk of combined CV events in RA patients with hypercholesterolaemia versus those without hypercholesterolaemia using random effect model. (TIFF)

S5 Fig Forest plot depicting the relative risk of combined CV events in obese RA patients versus s those without obesity using random effect model. (TIFF)

S6 Fig Forest plot depicting the relative risk of combined CV events in physical inactive RA patients versus physical active using random effect model. (TIFF)

S1 Materials PRISMA 2009 flow diagram. (PDF)

S2 Materials PRISMA 2009 checklist. (DOC)

S3 Materials Appendix 1. (DOCX)

S1 Table Checklist for assessing quality of included studies in the meta-analysis. (DOCX)

S2 Table Excluded studies and reasons of exclusion. (DOCX)

S3 Table Exposures and outcomes in studies included in the meta-analysis. (DOCX)

S4 Table Effect estimates of myocardial infarction in RA patients compared to general population. (DOCX)
