# Supplementary figures and images for: The Impact of Traditional Cardiovascular Risk Factors on Cardiovascular Outcomes in Patients with Rheumatoid Arthritis: A Systematic Review and Meta-Analysis
Source: PLoS One. 2015 Feb 17;10(2):e0117952. doi: 10.1371/journal.pone.0117952 (PMC4331556; doi:10.1371/journal.pone.0117952)

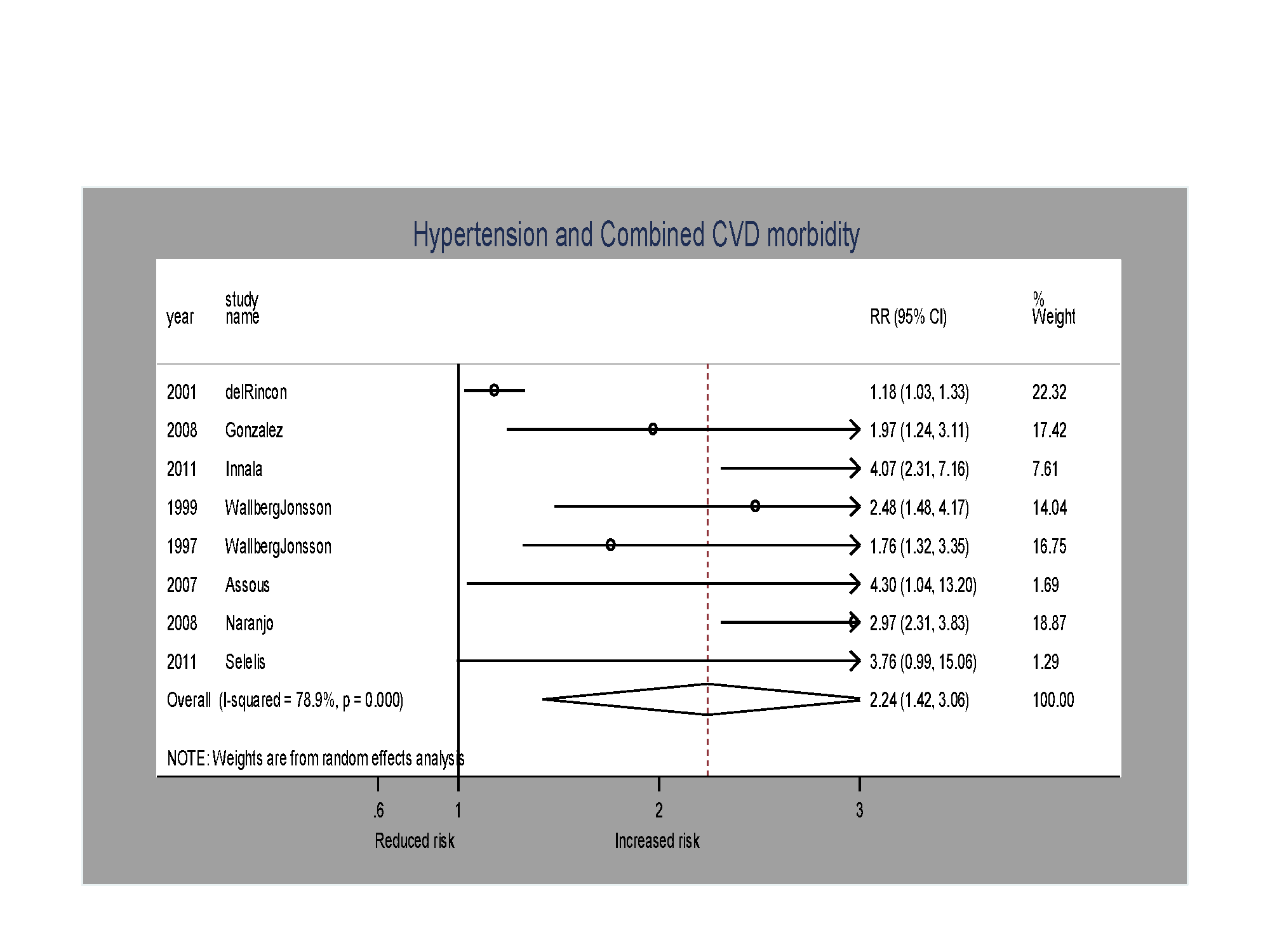

Supplement: S1 Fig — (TIFF) [file pone.0117952.s001.tiff]

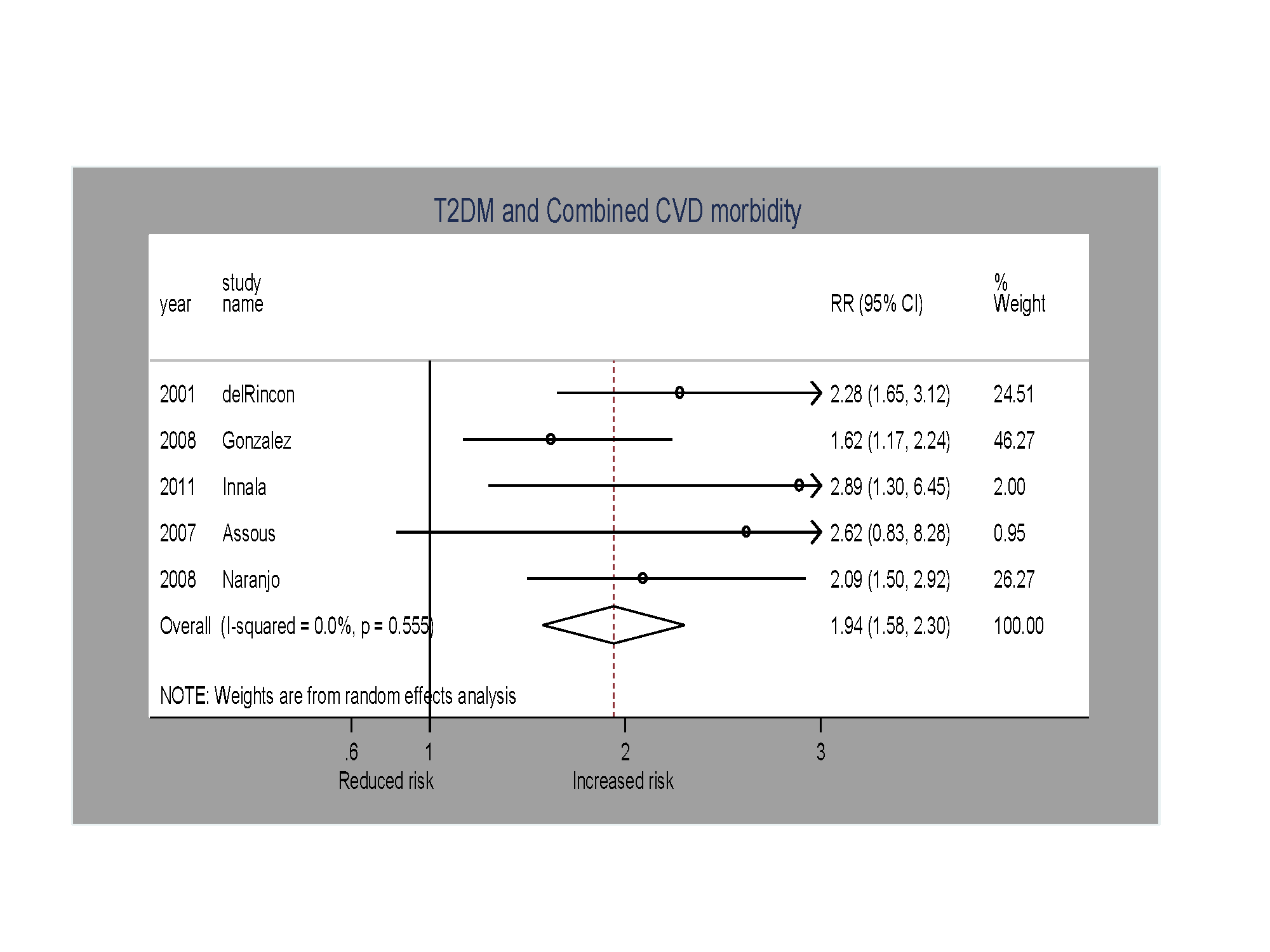

Supplement: S2 Fig — (TIFF) [file pone.0117952.s002.tiff]

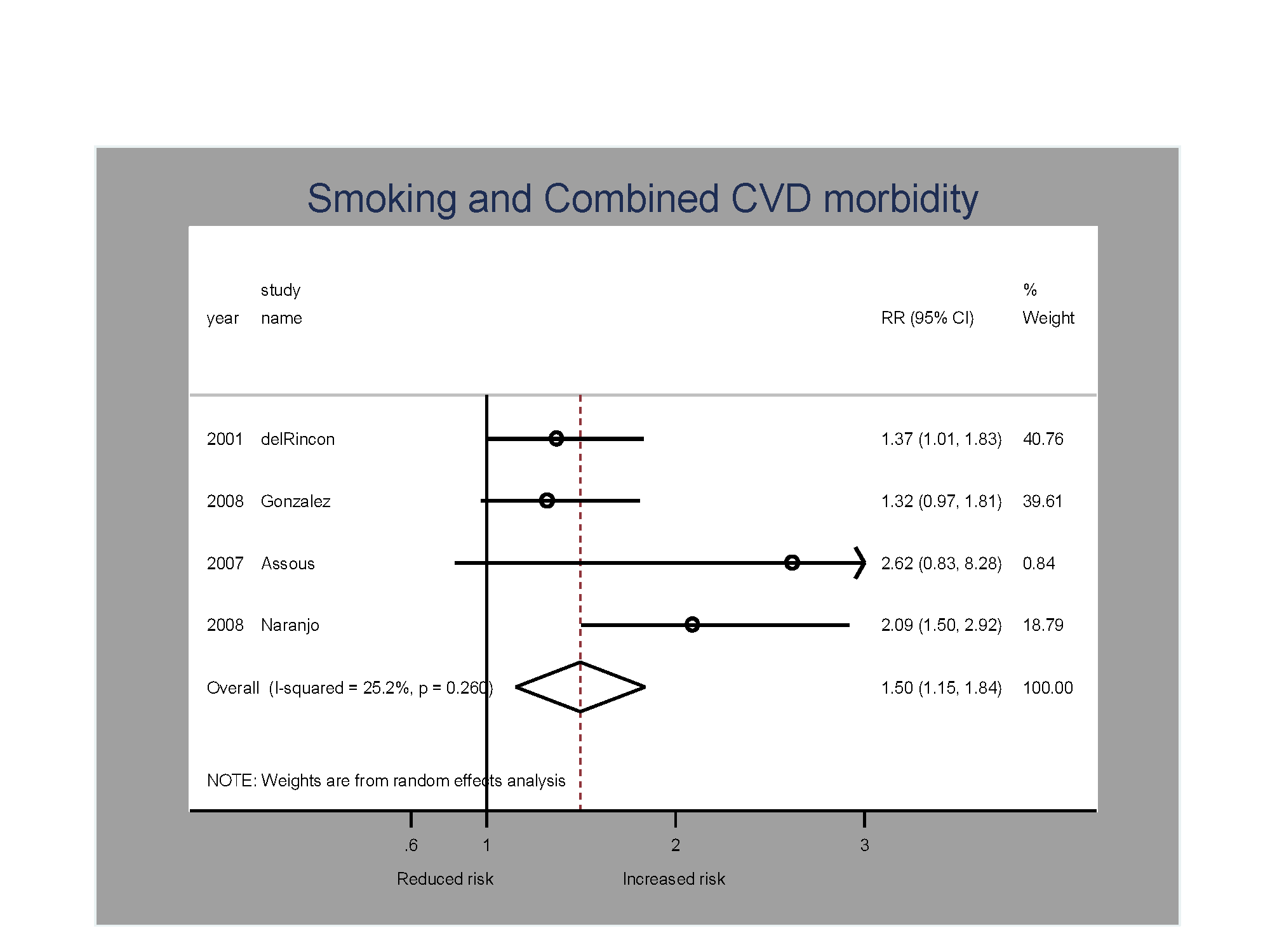

Supplement: S3 Fig — (TIFF) [file pone.0117952.s003.tiff]

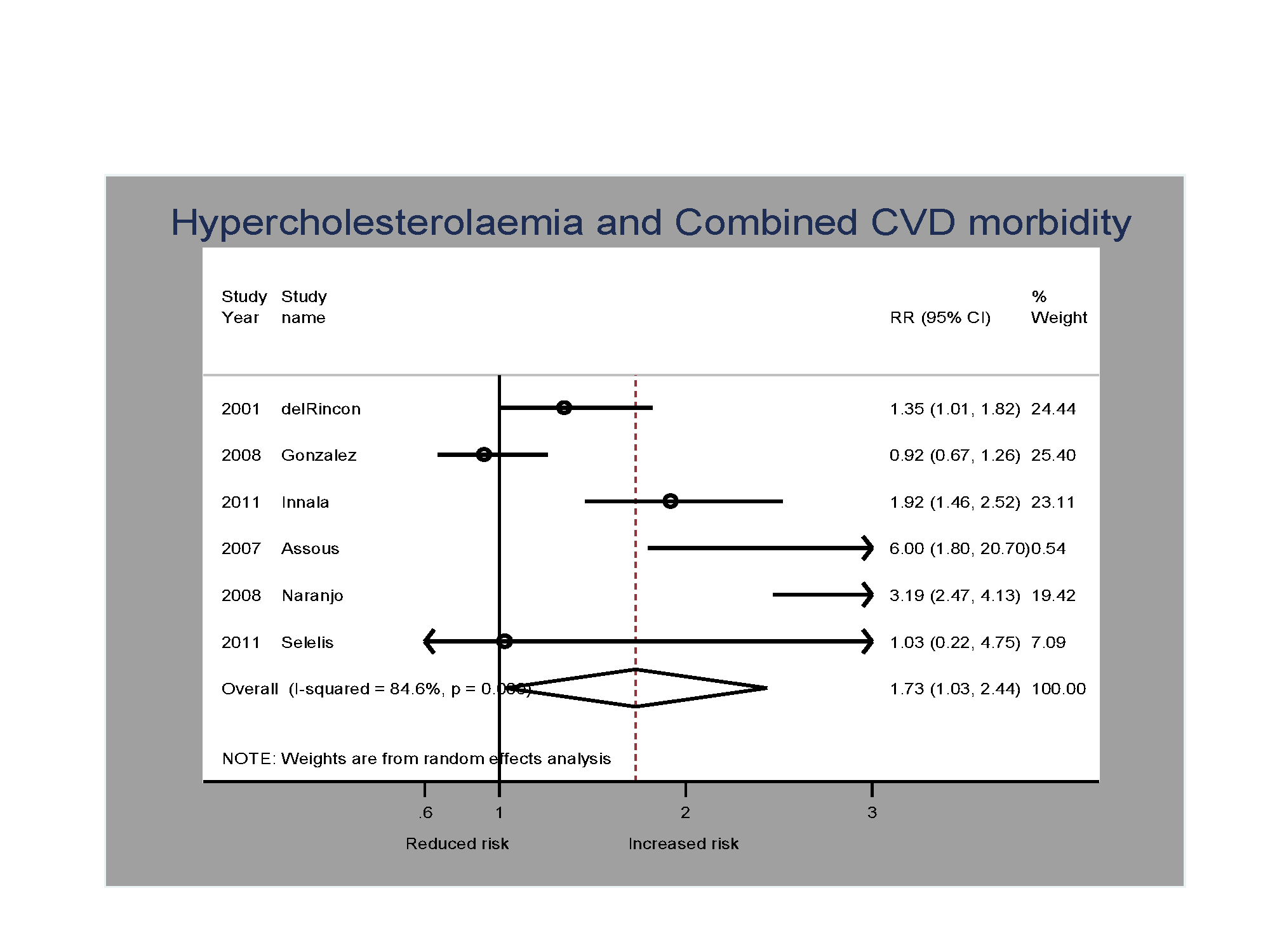

Supplement: S4 Fig — (TIFF) [file pone.0117952.s004.tiff]

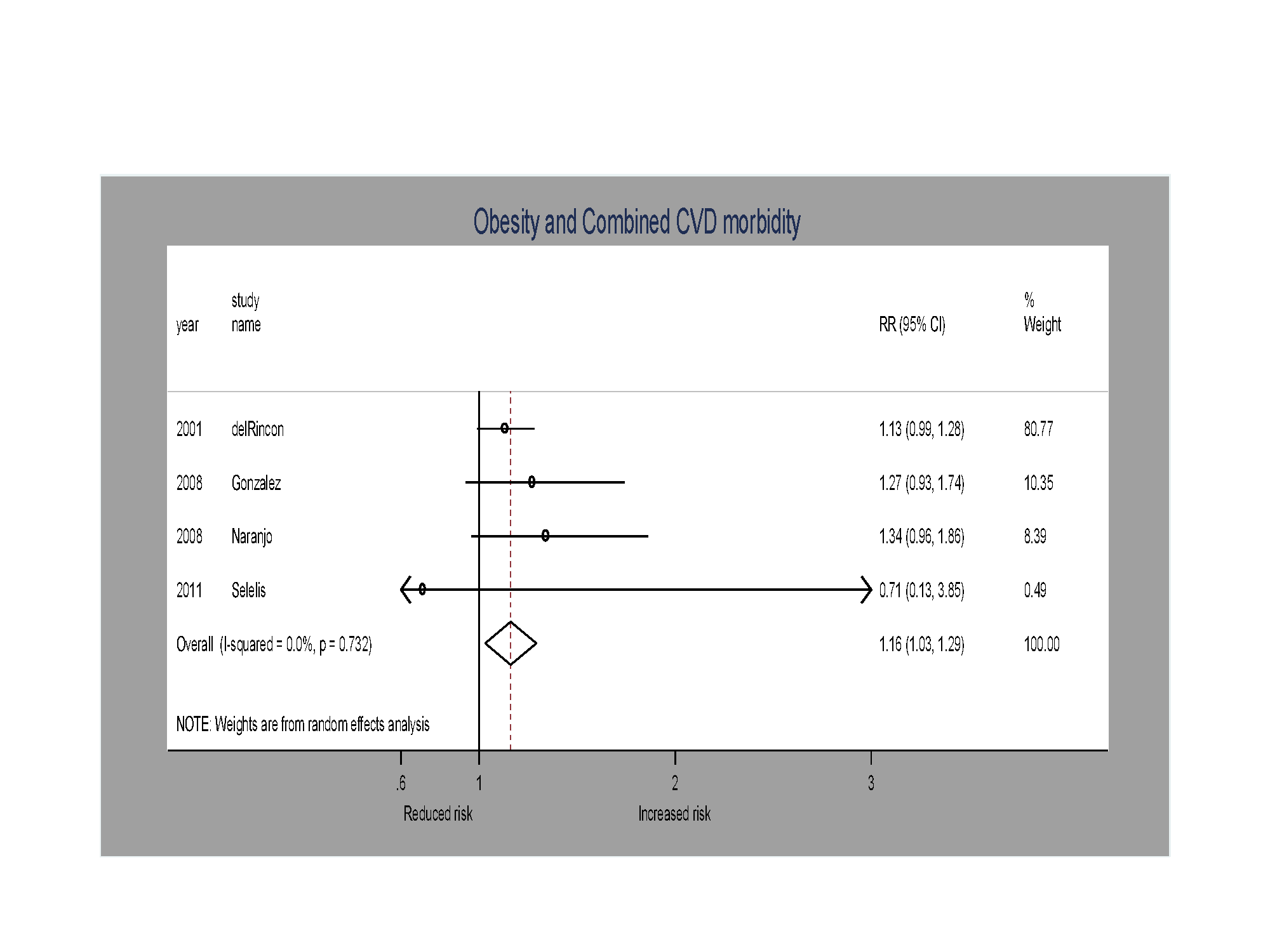

Supplement: S5 Fig — (TIFF) [file pone.0117952.s005.tiff]

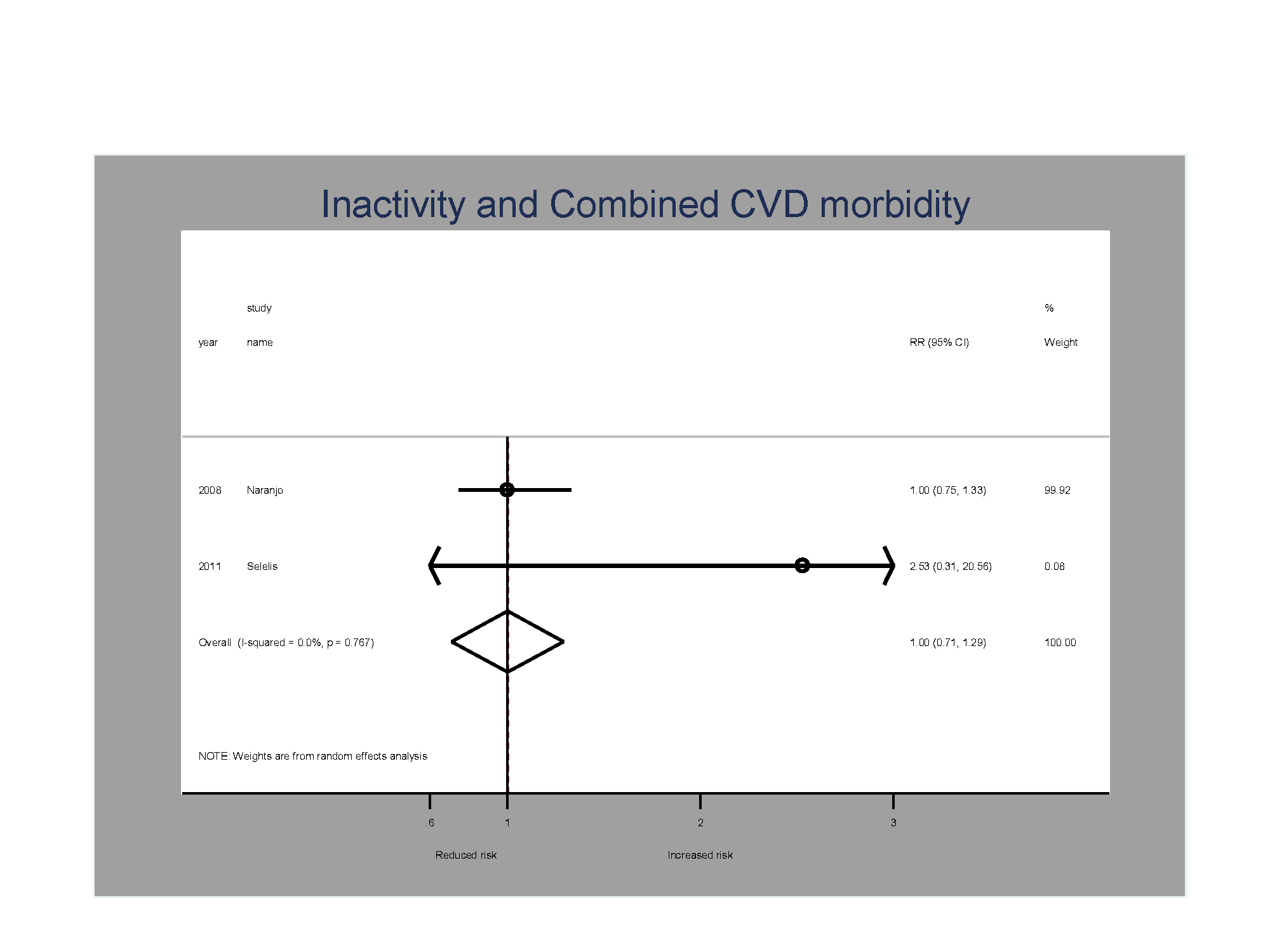

Supplement: S6 Fig — (TIFF) [file pone.0117952.s006.tiff]
