# Supplementary material for: The Impact of Traditional Cardiovascular Risk Factors on Cardiovascular Outcomes in Patients with Rheumatoid Arthritis: A Systematic Review and Meta-Analysis
Source: PLoS One. 2015 Feb 17;10(2):e0117952. doi: 10.1371/journal.pone.0117952 (PMC4331556; doi:10.1371/journal.pone.0117952)
Supplement: S3 Materials — (DOCX) [file pone.0117952.s009.docx]

**Appendix 1.** **Data sources**

**PubMed**

((arthriti* AND rheumatoid) OR Arthritis, Rheumatoid[Mesh]) AND ((Cardiovascular Diseases[Mesh] OR heart OR cardiac OR cardiovascular OR myocardial OR Myocardial Infarction[mesh] OR Stroke[MeSH] OR Cerebrovascular Accident OR Cerebrovascular Stroke OR cerebral vascular accident OR CVA OR endothelial dysfunction OR vascular stiffness[mesh] OR arterial stiffness OR aortic stiffness OR Peripheral Arterial Disease[Mesh] OR Peripheral Arterial Disease) AND (Risk[Mesh] OR Risk Assessment[Mesh] OR Risk Management[Mesh] OR Risk Factors[Mesh] OR risk OR risks OR outcome* OR event* OR complication* OR morbidit* OR mortalit* OR hypertensi* OR hypertension[mesh] OR Hypercholesterolemia[mesh] OR ((LDL OR HDL)) AND cholesterol) OR smoking[mh:noexp] OR smok8 OR Diabetes Mellitus, Type 2[mh] OR (diabetes AND (type 2 OR type II)) OR overweight[mh] OR obes* OR body mass index[mesh] OR body mass index)) AND English[la]

**Scopus**

(arthriti* AND rheumatoid)

AND

((heart OR cardiac OR cardiovascular OR cardio-vascular OR myocardial OR Stroke OR “Cerebrovascular Accident” OR “Cerebrovascular Stroke” OR “cerebral vascular accident” OR CVA OR “endothelial dysfunction” OR “vascular stiffness” OR “arterial stiffness” OR “aortic stiffness” OR “Peripheral Arterial Disease”)

AND

(Risk* OR outcome* OR event* OR complication* OR morbidit* OR mortalit* OR hypertensi* OR Hypercholesterolemia OR ((LDL OR HDL) AND cholesterol) OR smok* OR (diabetes AND (“type 2” OR “type II”)) OR overweight OR obes* OR body mass index))

AND

(patient* OR person* OR human* OR subjects OR male* OR female*)

**Cochrane Library**

#1 MeSH descriptor: [Arthritis, Rheumatoid] explode all trees

#2 (arthriti* and rheumatoid)

#3 #1 or #2

#4 MeSH descriptor: [Cardiovascular Diseases] explode all trees

#5 MeSH descriptor: [Myocardial Infarction] explode all trees

#6 MeSH descriptor: [Stroke] explode all trees

#7 MeSH descriptor: [Vascular Stiffness] explode all trees

#8 MeSH descriptor: [Peripheral Arterial Disease] explode all trees

#9 heart or cardiac or cardiovascular or myocardial

#10 stroke or cerebrovascular accident or cerebral vascular accident or CVA

#11 endothelial dysfunction or arterial stiffness or aortic stiffness or peripheral arterial disease

#12 #4 or #5 or #6 or #7 or #8 or #9 or #10 or #11

#13 MeSH descriptor: [Risk] explode all trees

#14 MeSH descriptor: [Risk Assessment] explode all trees

#15 MeSH descriptor: [Risk Management] explode all trees

#16 MeSH descriptor: [Risk Factors] explode all trees

#17 risk* or outcome* or event* or complication* or morbidit* or mortalit*

#18 MeSH descriptor: [Hypertension] explode all trees

#19 MeSH descriptor: [Hypercholesterolemia] explode all trees

#20 MeSH descriptor: [Smoking] explode all trees

#21 MeSH descriptor: [Diabetes Mellitus, Type 2] explode all trees

#22 MeSH descriptor: [Overweight] explode all trees

#23 MeSH descriptor: [Body Mass Index] explode all trees

#24 hypertensi* or ((LDL or HDL) and cholesterol) or smok* or (diabetes and (type 2 or type II)) or obes* or body mass index

#25 #13 or #14 or #15 or #16 or #17 or #18 or #19 or #20 or #21 or #22 or #23 or #24

#26 #3 and #12 and #25 463
