# Supplementary material for: The Impact of Traditional Cardiovascular Risk Factors on Cardiovascular Outcomes in Patients with Rheumatoid Arthritis: A Systematic Review and Meta-Analysis
Source: PLoS One. 2015 Feb 17;10(2):e0117952. doi: 10.1371/journal.pone.0117952 (PMC4331556; doi:10.1371/journal.pone.0117952)
Supplement: S2 Table — (DOCX) [file pone.0117952.s011.docx]

**Table S2**. **Excluded studies and reasons of exclusion**

| Author, publication year | Title | Reason of exclusion |
| --- | --- | --- |
| (Del Rincon et al. 2007) | Acceleration of atherosclerosis during the course of  rheumatoid arthritis | No information was available for CV risk factors effect on CV event |
| (Chung et al. 2012) | Prevalence of traditional modifiable cardiovascular risk factors in patients with rheumatoid arthritis: comparison with control subjects from the multi-ethnic study of atherosclerosis | No information were available for CV risk factors effect on CV event |
| (Boyer et al. 2011) | Traditional cardiovascular risk factors in rheumatoid arthritis: a meta-analysis | Not fulfilled the inclusion criteria (review and references were hand searched for relevant studies) |
| (Burnner et al. 2006) | Ankylosing spondylitis and heart abnormalities: do cardiac conduction disorders, valve regurgitation and diastolic dysfunction occur more often in male patients with diagnosed ankylosing spondylitis for over 15 years than in the normal population? | No information was available for CV risk factors effect on CV event |
| (Kishimoto et al. 2009) | Arthritis as a risk factor for incident coronary heart disease in elderly Japanese-American males - the Honolulu Heart Program | Not fulfilled the inclusion criteria |
| (Giles et al. 2010) | Abdominal adiposity in rheumatoid arthritis: association with cardiometabolic risk factors and disease characteristics | Not fulfilled the inclusion criteria |
| (Onuora 2012) | Rheumatoid arthritis: How bad is obesity for RA? | No information was available for CV risk factors effect on CV event |
| (Agarwal and Malaviya 2013) | A study of the conventional cardiovascular disease (CVD) risk factors in rheumatoid arthritis (RA) among Indians | No information was available for CV risk factors effect on CV event |
| (Nurmohamed et al. 2012) | Cardiovascular and cerebrovascular diseases in ankylosing spondylitis: current insights | Not fulfilled the inclusion criteria |
| (Kaplan 2006) | Cardiovascular disease in rheumatoid arthritis | Not fulfilled the inclusion criteria (review and references were hand searched for relevant studies) |
| (Mathieu et al. 2011) | Cardiovascular profile in ankylosing spondylitis: a systematic review and meta-analysis | Not fulfilled the inclusion criteria (review and references were hand searched for relevant studies) |
| (McEntegart et al. 2001) | Cardiovascular risk factors, including thrombotic variables, in a population with rheumatoid arthritis | No information was available for CV risk factors effect on CV event |
| (Mathieu et al. 2010) | Spondyloarthropathies: an independent cardiovascular risk factor? | Not fulfilled the inclusion criteria |
| (del Rincon et al. 2005) | Relative contribution of cardiovascular risk factors and rheumatoid arthritis clinical manifestations to atherosclerosis. | No information about clinical CV outcomes |
| (Chung et al. 2005) | Increased coronary-artery atherosclerosis in rheumatoid arthritis: relationship to disease duration and cardiovascular risk factors | No information about clinical CV outcomes |
| (Hamdi et al. 2012) | Assessment of preclinical atherosclerosis in patients with ankylosing spondylitis. | No information about clinical CV outcomes |
| (Pahor et al. 2006) | Accelerated atherosclerosis in pre-menopausal female patients with rheumatoid arthritis. | No information about clinical CV outcomes |
| (Sodergren et al. 2010) | Atherosclerosis in early rheumatoid arthritis: very early endothelial activation and rapid progression of intima media thickness | No information about clinical CV outcomes |
| (Cypiene et al. 2010) | The influence of mean blood pressure on arterial stiffening and endothelial dysfunction in women with rheumatoid arthritis and systemic lupus erythematosus | No information about clinical CV outcomes |
| (Zampeli et al. 2012) | Predictors of new atherosclerotic carotid plaque development in patients with rheumatoid arthritis: a longitudinal study | No information about clinical CV outcomes |
| (Gabriel and Crowson 2012) | Risk factors for cardiovascular disease in rheumatoid arthritis | Not fulfilled the inclusion criteria (review and references were hand searched for relevant studies) |
| (Peters et al. 2010) | Signs of accelerated preclinical atherosclerosis in patients with ankylosing spondylitis | No information about clinical CV outcomes |
| (Dessein et al. 2005) | Traditional and nontraditional cardiovascular risk factors are associated with atherosclerosis in rheumatoid arthritis | No information about clinical CV outcomes |
| (Solomon et al. 2012) | The carotid artery atherosclerosis burden and its relation to cardiovascular risk factors in black and white Africans with established rheumatoid arthritis: a cross-sectional study | No information about clinical CV outcomes |
| (Ahmad et al. 2012) | Predictors of atherosclerosis in rheumatoid arthritis | No information about clinical CV outcomes |
| (Azevedo and Pecoits-Filho 2010) | Atherosclerosis and endothelial dysfunction in patients with ankylosing spondylitis | No information about clinical CV outcomes |
| (Chatterjee et al. 2012) | Subclinical atherosclerosis and endothelial dysfunction in patients with early rheumatoid arthritis as evidenced by measurement of carotid intima-media thickness and flow-mediated vasodilatation: an observational study | No information about clinical CV outcomes |
| (Crilly and McNeill 2012) | Arterial dysfunction in patients with rheumatoid arthritis and the consumption of daily fruits and daily vegetables | No information about clinical CV outcomes |
| (Dessein et al. 2005) | Biomarkers of endothelial dysfunction, cardiovascular risk factors and atherosclerosis in rheumatoid arthritis | No information about clinical CV outcomes |
| (Dessein et al. 2007) | Influence of nonclassical cardiovascular-risk factors on the accuracy of predicting subclinical atherosclerosis in rheumatoid arthritis | No information about clinical CV outcomes |
| (Evans et al. 2011) | Carotid atherosclerosis predicts incident acute coronary syndromes in rheumatoid arthritis | No information about clinical CV outcomes |
| (Fietta and Delsante 2009) | Atherogenesis in rheumatoid arthritis: the "rheumatoid vasculopathy"? | No information about clinical CV outcomes |
| (Gerli et al. 2007) | Precocious atherosclerosis in rheumatoid arthritis: role of traditional and disease-related cardiovascular risk factors | No information about clinical CV outcomes |
| (Gerli et al. 2005) | Early atherosclerosis in rheumatoid arthritis: effects of smoking on thickness of the carotid artery intima media | No information about clinical CV outcomes |
| (Giles et al. 2009) | Coronary arterial calcification in rheumatoid arthritis: Comparison with the Multi-Ethnic Study of Atherosclerosis | No information about clinical CV outcomes |
| (Jonsson et al. 2001) | Increased prevalence of atherosclerosis in patients with medium term rheumatoid arthritis | No information about clinical CV outcomes |
| (Huang et al. 2012) | Systolic blood pressure is a useful measure in predicting the risk of endothelial dysfunction in patients with rheumatoid arthritis | Not fulfilled the inclusion criteria |
| (Klocke et al. 2003) | Arterial stiffness and central blood pressure, as determined by pulse wave analysis, in rheumatoid arthritis | No information about clinical CV outcomes |
| (Inaba et al. 2007) | Independent association of increased trunk fat with increased arterial stiffening in postmenopausal patients with rheumatoid arthritis | No information about clinical CV outcomes |
| (Kim et al. 2012) | Implication of new atherosclerotic carotid plaques in the cardiovascular outcome of patients with rheumatoid arthritis | No information about clinical CV outcomes |
| (Nagata-Sakurai et al. 2003) | Inflammation and bone resorption as independent factors of accelerated arterial wall thickening in patients with rheumatoid arthritis | No information about clinical CV outcomes |
| (Pieringer et al. 2010) | Arterial stiffness in a muscular artery in women with longstanding rheumatoid arthritis compared with healthy controls and patients with traditional cardiovascular risk factors | No information about clinical CV outcomes |
| (Popa et al. 2012) | Atherogenic index and high-density lipoprotein cholesterol as cardiovascular risk determinants in rheumatoid arthritis: the impact of therapy with biologicals | No information about the impact of CV risk factors was studied. |
| (Roman et al. 2006) | Preclinical carotid atherosclerosis in patients with rheumatoid arthritis | No information about clinical CV outcomes |
| (Shakeri et al. 2011) | Common carotid intima-media thickness in patients with late rheumatoid arthritis; what is the role of gender? | No information about clinical CV outcomes |
| (Sliem and Nasr 2010) | Change of the aortic elasticity in rheumatoid arthritis: Relationship to associated cardiovascular risk factors | No information about clinical CV outcomes |
| (Solomon et al. 2010) | Risk factor profiles for atherosclerotic cardiovascular disease in black and other Africans with established rheumatoid arthritis | No information about clinical CV outcomes |
| (Solomon et al. 2012) | Obesity and carotid atherosclerosis in African black and Caucasian women with established rheumatoid arthritis: a cross-sectional study | No information about clinical CV outcomes |
| (Ajeganova et al. 2012) | Carotid atherosclerosis, disease measures, oxidized low-density lipoproteins, and atheroprotective natural antibodies for cardiovascular disease in early rheumatoid arthritis - An inception Cohort study | No information about clinical CV outcomes |
| (Alkaabi et al. 2003) | Rheumatoid arthritis and macrovascular disease | No information about clinical CV outcomes |
| (Alves et al. 1988) | Cardiac alterations in ankylosing spondylitis | No information was available for CV risk factors effect on CV event |
| (Arts et al. 2012) | High-density lipoprotein cholesterol subfractions HDL2 and HDL3 are reduced in women with rheumatoid arthritis and may augment the cardiovascular risk of women with RA: a cross-sectional study | No information about clinical CV outcomes |
| (Bell and Rowe 2011) | The recognition and assessment of cardiovascular risk in people with rheumatoid arthritis in primary care: a questionnaire-based study of general practitioners | Not fulfilled the inclusion criteria |
| (Chogle and Chakravarty 2007) | Cardiovascular events in systemic lupus erythematosus and rheumatoid arthritis : emerging concepts, early diagnosis and management | No information was available for CV risk factors effect on CV event |
| (Cisternas et al. 2002) | Cardiovascular risk factors in Chilean patients with rheumatoid arthritis | No information about clinical CV outcomes |
| (De Pablo et al. 2007) | Antioxidants and other novel cardiovascular risk factors in subjects with rheumatoid arthritis in a large population sample | No information about clinical CV outcomes |
| (Gabriel 2008) | Cardiovascular morbidity and mortality in rheumatoid arthritis | No information was available for CV risk factors effect on CV event |
| (Gabriel 2010) | Heart disease and rheumatoid arthritis: understanding the risks | No information was available for CV risk factors effect on CV event le |
| (Gerli and Goodson 2005) | Cardiovascular involvement in rheumatoid arthritis | No information was available for CV risk factors effect on CV event |
| (Goodson 2002) | Coronary artery disease and rheumatoid arthritis | No information was available for CV risk factors effect on CV event |
| (Han et al. 2006) | Cardiovascular disease and risk factors in patients with rheumatoid arthritis, psoriatic arthritis, and ankylosing spondylitis | No information about clinical CV outcomes |
| (Kawada 2013) | Prognosis factors for cardiovascular events in patients with rheumatoid arthritis: multivariate analysis is indispensable | Not fulfilled the inclusion criteria (it is a comment) |
| (Kumar and Armstrong 2008) | Cardiovascular disease--the silent killer in rheumatoid arthritis | No information was available for CV risk factors effect on CV event |
| (Liao and Solomon 2013) | Traditional cardiovascular risk factors, inflammation and cardiovascular risk in rheumatoid arthritis | No information was available for CV risk factors effect on CV event |
| (McEntegart et al. 2001) | Cardiovascular risk factors, including thrombotic variables, in a population with rheumatoid arthritis | No information was available about the clinical CV outcomes |
| (McLaren et al. 2002) | Activated factor XII in rheumatoid arthritis | No information was available for CV risk factors effect on CV event |
| (Panoulas et al. 2008) | Hypertension in rheumatoid arthritis | Not fulfilled the inclusion criteria (review and references were hand searched for relevant studies) |
| (Papagoras et al. 2013) | Atherosclerosis and cardiovascular disease in the spondyloarthritides, particularly ankylosing spondylitis and psoriatic arthritis | Not fulfilled the inclusion criteria |
| (Pemberton et al. 2009) | Biomarkers of oxidant stress, insulin sensitivity and endothelial activation in rheumatoid arthritis: a cross-sectional study of their association with accelerated atherosclerosis | No information about clinical CV outcomes |
| (Peters et al. 2004) | Cardiovascular risk profile of patients with spondylarthropathies, particularly ankylosing spondylitis and psoriatic arthritis | Not fulfilled the inclusion criteria |
| (Pieringer et al. 2012) | Rheumatoid arthritis is an independent risk factor for an increased augmentation index regardless of the coexistence of traditional cardiovascular risk factors | No information about clinical CV outcomes |
| (Saravana and Gillott 2004) | Ischaemic heart disease in rheumatoid arthritis patients | Not fulfilled the inclusion criteria (letters to the Editor) |
| (Watson et al. 2003) | All-cause mortality and vascular events among patients with rheumatoid arthritis, osteoarthritis, or no arthritis in the UK General Practice Research Database | No information was available for CV risk factors effect on CV event |
| (Wasko 2008) | Rheumatoid arthritis and cardiovascular disease | No information was available for CV risk factors effect on CV event |
| (Stamatelopoulos et al. 2010) | Subclinical peripheral arterial disease in rheumatoid arthritis | No information about clinical CV outcomes |
| (Solomon et al. 2010) | Explaining the cardiovascular risk associated with rheumatoid arthritis: traditional risk factors versus markers of rheumatoid arthritis severity | No information was available for each CV risk factor separately |
| (Steen et al. 2009) | High incidence of cardiovascular events in patients with rheumatoid arthritis | No information was available for CV risk factors effect on CV event |
| (Symmons and Gabriel 2011) | Epidemiology of CVD in rheumatic disease, with a focus on RA and SLE | Not fulfilled the inclusion criteria |
| (van Halm et al. 2006) | Disease-modifying antirheumatic drugs are associated with a reduced risk for cardiovascular disease in patients with rheumatoid arthritis: a case control study | No information was available for CV risk factors effect on CV event |
| (Vinsonneau et al. 2008) | Cardiovascular disease in patients with spondyloarthropathies | Not fulfilled the inclusion criteria |
| (Wolfe and Michaud 2012) | Effect of body mass index on mortality and clinical status in rheumatoid arthritis | No information about clinical CV outcomes |
| (Maradit-Kremers et al. 2005) | Increased unrecognized coronary heart disease and sudden deaths in rheumatoid arthritis: a population-based cohort study | The outcome is sudden death and the same population sample was used by the same author in another study |
| (Maradit-Kremers et al. 2005) | Cardiovascular death in rheumatoid arthritis | Same population sample was used by the same author in another study |
| (Kremers et al. 2004) | Prognostic importance of low body mass index in relation to cardiovascular mortality in rheumatoid arthritis | Same population sample was used by the same author in another study |
| (Brophy et al. 2012) | No increased rate of acute myocardial infarction or stroke among patients with ankylosing spondylitis-a retrospective cohort study using routine data | No information was available for CV risk factors effect on CV event |
| (Metsios et al. 2009) | Rheumatoid cachexia and cardiovascular disease | Not fulfilled the inclusion criteria |
| (Solomon et al. 2003) | Cardiovascular morbidity and mortality in women diagnosed with rheumatoid arthritis | No information was available for CV risk factors effect on CV event |
| (Solomon et al. 2004) | Cardiovascular risk factors in women with and without rheumatoid arthritis | No information was available for CV risk factors effect on CV event |
| (Singh et al. 2003) | Consequences of increased systolic blood pressure in patients with osteoarthritis and rheumatoid arthritis | No information was available for CV risk factors effect on CV event |
| (Semb et al. 2012) | Prediction of cardiovascular events in patients with ankylosing spondylitis and psoriatic arthritis: role of lipoproteins in a high-risk population | Not fulfilled the inclusion criteria |
| (Nicola et al. 2005) | The risk of congestive heart failure in rheumatoid arthritis: a population-based study over 46 years | Not fulfilled the inclusion criteria |
| (Crowson et al. 2005) | How much of the increased incidence of heart failure in rheumatoid arthritis is attributable to traditional cardiovascular risk factors and ischemic heart disease? | Not fulfilled the inclusion criteria |
| (Hamdi et al. 2012) | Assessment of preclinical atherosclerosis in patients with ankylosing spondylitis | No information about clinical CV outcomes |
| (Ajeganova et al. 2013) | Association of obesity with worse disease severity in rheumatoid arthritis as well as with comorbidities: A long-term follow-up from disease onset | The required data was not available |
| (Mohammad et al. 2010) | Increased occurrence of cardiovascular events and comorbidities in a general rheumatology cohort | The required data was not available |
| (Metsios et al. 2009) | Association of physical inactivity with increased cardiovascular risk in patients with rheumatoid arthritis | The required data was not available |
| (Brady et al. 2009) | The role of traditional cardiovascular risk factors among patients with rheumatoid arthritis | The required data was not available |
| (Kremers et al. 2008) | High ten-year risk of cardiovascular disease in newly diagnosed rheumatoid arthritis patients: a population-based cohort study | The required data was not available |
| (Nadareishvili et al. 2008) | Cardiovascular, rheumatologic, and pharmacologic predictors of stroke in patients with rheumatoid arthritis: a nested, case-control study | The required data was not available |

IMT= Carotid Intima-Media Thickness, CHF=Congestive Heart Failure, CV= Cardiovascular, CVD= Cardiovascular Disease, RA= Rheumatoid Arthritis
