# Supplementary material for: The Impact of Traditional Cardiovascular Risk Factors on Cardiovascular Outcomes in Patients with Rheumatoid Arthritis: A Systematic Review and Meta-Analysis
Source: PLoS One. 2015 Feb 17;10(2):e0117952. doi: 10.1371/journal.pone.0117952 (PMC4331556; doi:10.1371/journal.pone.0117952)
Supplement: S3 Table — (DOCX) [file pone.0117952.s012.docx]

**Table S3. Exposures and outcomes in studies included in the meta-analysis**

| Reference | Risk factors included | CV outcomes | Type of data |
| --- | --- | --- | --- |
| [47] | Hypertension, T2D, Smoking | MI | Raw data |
| [21] | Hypertension, T2D | MI | Raw data |
| [10] | Hypertension, T2D, *Smoking, *Hypercholesterolaemia | MI as a separate outcome | Raw data |
|  |  | Combined CV morbidity: MI (as a combined outcome), ischemic stroke, and congestive heart failure | Raw data; Effect sizes and CI for hypercholesterolaemia |
| [36] | Hypertension, T2D, Smoking, Hypercholesterolaemia, Obesity, Physical inactivity | Combined CV morbidity: MI, angina pectoris, coronary disease, and stroke | Raw data; Effect sizes and CI for hypercholesterolaemia, obesity and physical inactivity |
| [15] | Hypertension, Hypercholesterolaemia, Obesity, Physical inactivity | Combined CV morbidity: MI, angina pectoris, heart failure, stroke, peripheral arterial disease | Raw data; Effect sizes and CI for hypercholesterolaemia, obesity and physical inactivity |
| [19] | Hypertension, T2D, Smoking, Hypercholesterolaemia, Obesity | Combined CV morbidity: MI, stroke, other arterial occlusive events and arterial revascularization procedures | Effect sizes and CI |
| [25] | Hypertension, T2D, Smoking, Hypercholesterolaemia, Obesity | Combined CV morbidity: MI and CHF | Effect sizes and CI |
| [12] | Hypertension, T2D, Hypercholesterolaemia | Combined CV morbidity: MI, stroke and peripheral vascular disease | Effect sizes and CI |
| [48] | Hypertension | Combined CV morbidity: MI, peripheral vascular disease and stroke | Effect sizes and CI |
| [17] | Hypertension | Combined CV morbidity: MI, peripheral vascular disease and stroke | Effect sizes and CI |

*Data was available for combined CV morbidity only
