# Supplementary material for: The Impact of Traditional Cardiovascular Risk Factors on Cardiovascular Outcomes in Patients with Rheumatoid Arthritis: A Systematic Review and Meta-Analysis
Source: PLoS One. 2015 Feb 17;10(2):e0117952. doi: 10.1371/journal.pone.0117952 (PMC4331556; doi:10.1371/journal.pone.0117952)
Supplement: S4 Table — (DOCX) [file pone.0117952.s013.docx]

**Table S4. Effect estimates of myocardial infarction in RA patients compared to general population**

| CV risk factor | **RA patients** | ***General population** |
| --- | --- | --- |
|  | **Relative risk (95% CI)** **Odds ratio (99% CI)** | |
| Hypertension | 1.84 (95% CI 1.38, 2.46) | 1.91 (99% CI 1.74, 2.10) |
| Type 2 diabetes | 1.89(95% CI 1.36, 2.63) | 2.37 (99% CI 2.07, 2.71) |

* Adopted from [50]
